# Does combined ACL primary augmentation with autograft reconstruction reduces risk of failure at 3‐years follow‐up compared to ACL reconstruction?

**DOI:** 10.1002/jeo2.70886

**Published:** 2026-08-10

**Authors:** Corentin Herce, Nicolas Bouguennec, Philippe Colombet

**Affiliations:** ^1^ Clinique du Sport de Bordeaux Mérignac Mérignac France

**Keywords:** anterior cruciate ligament, augmentation, functional results, knee, ligaments, repair

## Abstract

**Purpose:**

Re‐rupture after anterior cruciate ligament (ACL) repair is frequent, raising the question of graft integration failure. However, the introduction of new materials and techniques has brought ACL repair back into fashion, especially ACL augmentation using the hamstrings. Purpose To evaluate ACL augmentation, using a hamstring graft, complete remnant conservation and its fixation to the femur, and to compare it with “conventional” ACL reconstruction with a semi‐tendinosus graft without anterolateral ligament reconstruction. The re‐rupture rate, re‐intervention rate, intraoperative data (tunnel size, meniscal lesions, tourniquet time), and post‐operative subjective scores (SKV, IKDC, ACL‐RSI) at 3 years follow‐up were compared for the two techniques.

**Methods:**

ACL augmentation was performed in 60 consecutive patients, 47 of whom were evaluated (ACL‐A group) (seven excluded for re‐rupture and six lost to follow‐up). This group was compared with a control group of 63 patients who had undergone “conventional” ACL reconstruction (ACL‐R group) with a semi‐tendinosus graft without anterolateral ligament reconstruction (three patients were excluded due to re‐rupture). Both groups were comparable for sex, age, body mass index, surgical delay, tunnel size and meniscal lesion rate.

**Results:**

There was no significant difference between the two groups for clinical outcomes at 3 years follow‐up: median scores for ACL‐RSI (ACL‐A: 75 vs. ACL‐R: 72; *p* = 0.27), IKDC (ACL‐A: 90 vs. ACL‐R: 86; *p* = 0.20), Tegner (ACL‐A: 7.0 vs. ACL‐R: 6.0; *p* = 0.19) or SKV (ACL‐A: 90 vs. ACL‐R: 90; *p* = 0.58). Graft failure rate was also similar at 3 years follow‐up (ACL‐A: 13.0% vs. ACL‐R: 4.8%; *p* = 0.18), so was complication rate (ACL‐A: 16.7% vs. ACL‐R: 11.1%; *p* = 0.44). The ACL‐A group had a significantly longer median tourniquet time (42 min) compared to the ACL‐R group (35 min) (*p* < 0.0001).

**Conclusion:**

ACL augmentation did not improve clinical results, complication rates, or graft failure rate at 3 years post‐surgery, despite a longer surgery time and more challenging technique.

**Level of Evidence:**

Level II.

**Abbreviations**
ACL‐A: anterior cruciate ligament augmentation
ACL‐R: anterior cruciate ligament reconstruction

## INTRODUCTION

Anterior cruciate ligament (ACL) reconstruction is one of the most common procedures for knee ligament injuries, with between 100,000 and 200,000 cases performed annually in the United States [28]. However, graft failure remains frequent, most often due to insufficient graft integration [1]. Several techniques have therefore been developed to improve graft incorporation. Preserving the native ACL remnant may improve graft integration and clinical outcomes without increasing complication or cyclops rates [18, 29, 33].

ACL repair has theoretical advantages over reconstruction, including preservation of native biomechanics and proprioception, as well as decreased graft morbidity. Early results were initially promising but ACL primary repair was progressively abandoned because of high failure rates [8, 11]. More recently, ACL repair has regained interest with the development of modern arthroscopic techniques and rehabilitation protocols [16, 19]. Patient selection has also evolved, favouring proximal tears, good tissue quality, early surgery, and lower sport activity levels [27, 30].

Several augmentation procedures have been proposed to improve ACL repair outcomes, including dynamic intra‐ligamentary stabilisation [17], bioactive scaffold techniques [22], synthetic internal bracing [14] and biological augmentation [9, 10]. Few studies have directly compared ACL augmentation procedures with standard ACL reconstruction.

Colombet et al. described a technique combining ACL repair and reconstruction using a semitendinosus graft and the same femoral fixation device [4]. The aim of this technique is to preserve and tension the remnant around the graft in order to potentially improve graft incorporation and biological healing.

The purpose of this study was to compare ACL augmentation with standard ACL reconstruction using a hamstring graft. The primary outcome was post‐operative patient‐reported functional outcomes evaluated using the International Knee Documentation Committee (IKDC), ACL Return to Sport after Injury (ACL‐RSI), Tegner and Subjective Knee Value (SKV) scores at a minimum follow‐up of 3 years. Secondary outcomes included graft failure, reoperation rate, complications, and tourniquet time. We hypothesised that ACL augmentation could provide improved post‐operative functional outcomes and lower graft failure rates compared with standard ACL reconstruction.

## MATERIALS AND METHODS

### Study design and patients

This was a retrospective cohort, single centre study. All the patients were operated on by the same two senior surgeons and the surgical indications, fixation devices, follow‐up, and post‐operative protocol were similar for both groups.

The inclusion criteria for patients in the ACL augmentation group (ACL‐A group) were primary ACL augmentation, as described below, with >2 years follow‐up between January 2019 and October 2021. The inclusion criteria for patients undergoing ACL reconstruction (ACL‐R group) were ACL reconstruction with a semi‐tendinosus graft, with a minimum follow‐up of 3 years during the same period. We did not set a time limit between the injury and surgery.

The exclusion criteria were an anterolateral procedure, multi‐ligament surgery, associated bone surgery, or a lack of consent to inclusion in the study. In the ACL‐R group, patients were also excluded if they had a hamstring graft with both a semi‐tendinosus and gracilis tendon.

### Surgical techniques

For ACL reconstruction (ACL‐R), the technique used was similar to that described by Colombet et al. in 2015 [6]. The semi‐tendinosus was harvested and prepared in four strands with an endobutton PullUp (SBM®) for the femoral side and an endobutton PullUp XL (SBM®) for the tibial side. The femoral and tibial tunnel positions were identical in both groups and corresponded to a standard anatomic ACL reconstruction technique. The femoral tunnel was performed via an inside‐out technique with a 4.5 mm drill and then with a drill adapted to the diameter of the graft. A complete tibial tunnel was performed by an outside‐in technique. The graft was then passed through the tunnel and the adjustable devices were tightened.

**Figure 1:**
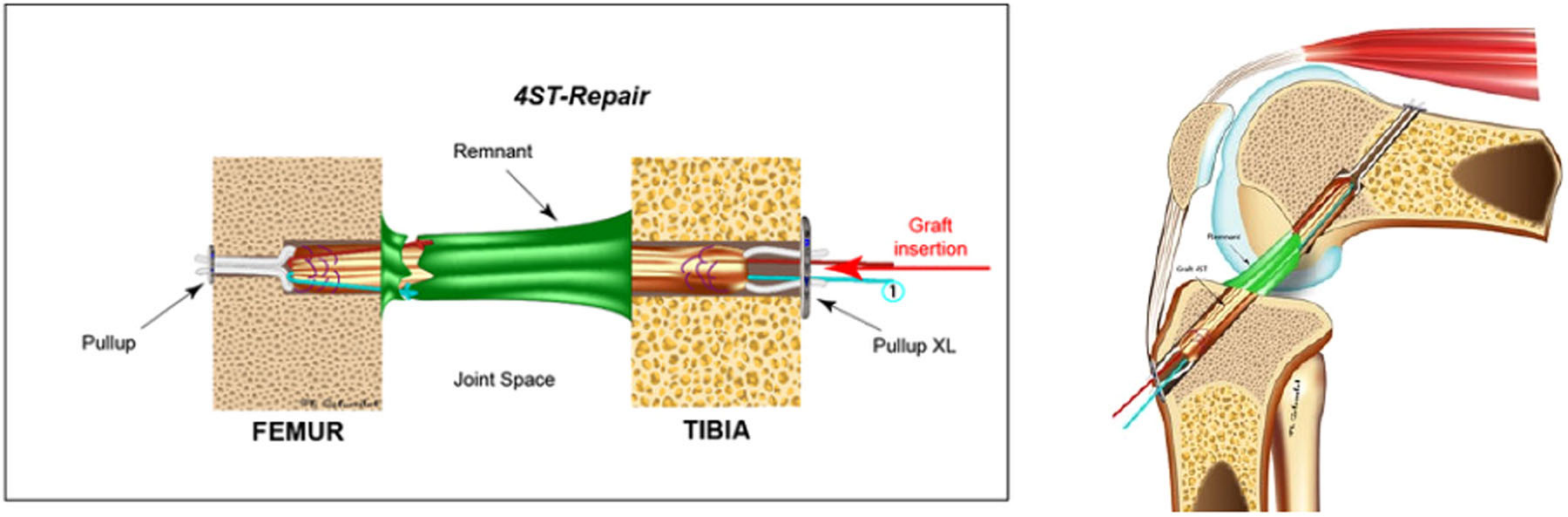
Anterior cruciate ligament (ACL) augmentation: The native ACL remnant is preserved, mobilised, and detached from its proximal adhesions. Two traction sutures are passed through the proximal remnant and retrieved through the tibial tunnel. Following anatomic semitendinosus ACL reconstruction, the traction sutures are secured through the femoral suspensory fixation device, allowing the remnant to be tensioned and wrapped around the graft while preserving its continuity and vascularises tissue.

In the ACL augmentation group (ACL‐A), the technique was similar to that described by Colombet et al. in 2020 [4].

he indication for ACL augmentation was made intraoperatively according to the remnant characteristics, following the principles described by Sherman et al. [27]. Augmentation was considered only in the presence of a repairable proximal tear (Sherman type I or selected type II tears), with good tissue quality, sufficient remnant length to allow tensioning to the femoral footprint, and satisfactory vascular appearance. Patients not meeting these criteria underwent standard ACL reconstruction. The posteromedial bundle was tested in Cabot's position [5]. The graft was prepared in the same manner as for the ACL‐R group. During the arthroscopic step, the entire remnant was preserved and detached from its upper adhesions to the femoral notch or posterior cruciate ligament. Two sutures were passed through the proximal part of the remnant using a Scorpion Suture Passer® (Arthrex). These sutures were then retrieved through the tibial tunnel in order to protect and tension the remnant during femoral tunnel drilling without damaging the remaining ACL fibres. Once the femoral tunnel was done, traction threads were passed in the loop of the femoral Pullup. The ACL graft was then passed and tightened, and the threads were tightened: the femoral endobutton served as a pulley. This step enabled the remnant to be mounted around the graft (Figure 1).

Surgery time were considered as equivalent to tourniquet time, as in our technique, we inflate the tourniquet before beginning the procedure and harvesting the graft, and deflate it after closing the incision.

The same rehabilitation protocol was followed in both groups, with an immediate full weight bearing and a full range of motion allowed. The only limitation was a flexion limitated to 90° during 6 weeks in case of posterior meniscal repair.

### Data collection

The following data were collected preoperatively: sex, age, body mass index (BMI), delay between trauma and surgery in month, tibial tunnel width, per operative meniscal lesions, tourniquet duration, complications and graft failure.

At the final follow‐up, the functional results were analysed using the IKDC, SKV and ACL‐RSI scores, evaluated during a dedicated consultation at > 3 years post‐surgery. The following were also evaluated as secondary end‐points: complication rate (post‐operative infections, graft rupture, reoperation rate) and tourniquet duration.

The study was conducted in accordance with national regulations and the principles of the Declaration of Helsinki and were assigned the IRB number CERC‐VS‐2025‐10‐1.

### Statistical analysis

Data are described as frequency and percentage for categorical variables, and median [interquartile range (25th–75th percentiles)] for quantitative variables. Group comparisons were conducted using the Student's t‐test or Mann–Whitney *U* test for continuous variables, and the Chi‐square test or Fisher's exact test for categorical variables, as appropriate. All tests were two‐sided, and statistical significance was set at *p* = 0.05. All analyses were performed using R open‐source software, version 3.4.4 (available online at http://www.Rproject.org). Patients with missing datas were excluded.

A post hoc analysis was performed for all study results. Relative risks (RRs) with 95% confidence intervals were calculated for graft failure and complication rates, whereas Cohen's *d* effect sizes were calculated for continuous variables. Post hoc power was estimated using two‐sided tests (*α* = 0.05) based on the observed differences between ACL‐A and ACL‐R groups.

## RESULTS

### ACL‐augmentation (ACL‐A) group

**Figure 2:**
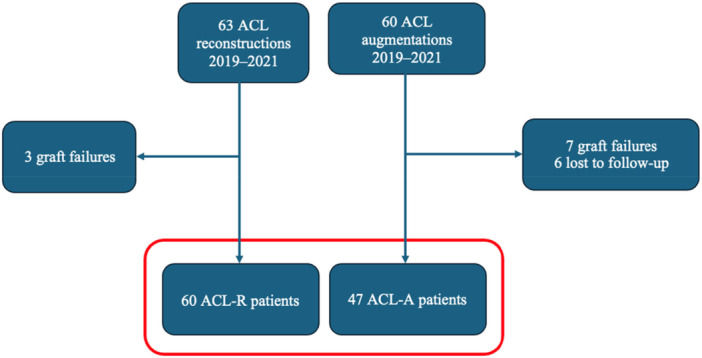
Flowchart of the study population. Between April 2019 and October 2021, 60 consecutive patients underwent anterior cruciate ligament (ACL) augmentation (ACL‐A) and 63 underwent standard ACL reconstruction (ACL‐R). In the ACL‐A group, seven patients experienced graft failure and six were lost to follow‐up, leaving 47 patients available for functional outcome assessment. In the ACL‐R group, three patients experienced graft failure, leaving 60 patients available for final evaluation.

Between April 2019 and October 2021, 60 patients underwent ACL augmentation (ACL‐A group (20 female and 40 male; median age 28 years [21; 39]). Median BMI was 23 kg/m^2^ [21; 25], and median tibial tunnel size was 8.5 mm [8.0; 9.0]. ACL‐A group had a median surgical delay of 7.6 month [0; 30]. Seven patients presented with graft failure and six were lost to follow‐up. These patients were excluded from the functional outcome results. Thus 47 patients were included for the final evaluation of functional outcomes (Figure 2). The demographic and clinical data for this group are summarised in Table 1.

**Table 1:** Demographic and clinical data for the study population.

Population	ACL‐A	ACL‐R	*p*‐Value
Sex (M/F)	20/40	35/28	0.144
Age (years)	28 [21; 39]	24 [18; 35]	0.057
BMI (kg/m^2^)	23 [21; 25]	23 [21; 26]	0.61
Surgery delay (month)	7.6 [0; 180]	5.0 [0; 120]	0.117
Tibial tunnel width	8.5 [8; 9]	9 [8.5; 9]	0.33
Meniscal lesion	21 (35%)	16 (25.4%)	0.33

*Note*: All values shown are median [IQR], or *n* (%). Comparison of patients undergoing ACL augmentation (ACL‐A) and standard ACL reconstruction (ACL‐R). Quantitative variables are presented as median [interquartile range], and categorical variables as number (%).

Abbreviations: ACL, anterior cruciate ligament; BMI, body mass index; IQR, interquartile range.

We also evaluated the clinical scores in the ACL‐A group with an mean ACL‐RSI score of 75 [56; 94], a mean IKDC of 90 [84; 97], a mean Tegner of 7 [4.8; 9] and a mean SKV of 90 [79; 100]. These functional scores are summarised in Table 2.

**Table 2:** Functional scores for the two groups of patients at 3‐year follow‐up.

Functional outcome	ACL‐A	ACL‐R	*p*‐Value
ACL‐RSI	75 [56; 94]	72 [51; 88]	0.27
IKDC	90 [84; 97]	86 [82; 94]	0.20
Tegner	7 [4.8; 9]	6 [4.5; 7]	0.19
SKV	90 [79; 100]	90 [80; 100]	0.58

*Note*: Data shown are median [IQR]. Comparison of post‐operative ACL‐RSI, IKDC, Tegner, and Subjective Knee Value (SKV) scores between the ACL augmentation (ACL‐A) and ACL reconstruction (ACL‐R) groups. Values are presented as median [IQR].

Abbreviations: ACL, anterior cruciate ligament; ACL‐RSI, ACL‐Return to Sport after Injury; IKDC, International Knee Documentation Committee; IQR, interquartile range.

### ACL‐R reconstruction (ACL‐R) group

During the period 2019–2021, 63 patients underwent ACL reconstruction using a semi‐tendinosus graft without anterolateral ligament reconstruction (35 female and 28 male; median age 24 years [18; 35]). Median BMI was 23 kg/m^2^ [21; 26], and median tibial tunnel size was 9.0 mm [8.5; 9]. ACL‐R group had a median surgical delay of 5 month [0; 31]. Three patients had graft failure and were excluded from the functional outcome results. Thus 60 patients were included in this group control for the functional outcomes (Figure 2). The demographic and clinical data for this group are summarised in Table 1.

We also evaluated the clinical scores in the ACL‐R group with an mean ACL‐RSI score of 72 [51; 88], a mean IKDC of 86 [82; 94], a mean Tegner of 6 [4.5; 7] and a mean SKV of 90 [80; 100]. These functional scores are summarised in Table 2.

### Functional results

Functional outcomes at 3‐year follow‐up were similar between groups for all evaluated PROMs (Table 2).

### Comparison of the two groups

The two groups were comparable regarding demographic and preoperative characteristics, including age, sex, BMI, delay between trauma and surgery, tunnel size and meniscal lesion rate (Table 1).

There was no significant difference in reoperation or graft failure rates between the two groups (Table 3).

**Table 3:** Failure rates and tourniquet time in the two groups of patients.

Results	ACL‐A	ACL‐R	*p*‐Value
Re‐operation	11 [20.7%]	7 [11.1%]	0.44
Graft failure	7 [13.0%]	3 [4.7%]	0.18
Tourniquet time (min)	42 [36; 46]	35 [25; 38]	<0.0001

*Note*: Data shown are median [IQR], or *n* (%). Comparison of reoperation rate, graft failure rate, and tourniquet time between the ACL augmentation (ACL‐A) and ACL reconstruction (ACL‐R) groups. Quantitative variables are presented as median [interquartile range], and categorical variables as number (%).

Abbreviations: ACL, anterior cruciate ligament; IQR, interquartile range.

There was no significant difference in graft failure rate (*p* = 0.18): seven (13.0%) in the ACL‐A group and three (4.7%) in the ACL‐R group. However, tourniquet time was significantly longer in the ACL‐A group (median 42 min [36; 46]) compared to ACL reconstruction (median 35 min [25; 38]) (*p* < 0.0001) (Table 3).

### Post hoc analysis

**Table 4:** Post hoc analysis.

Outcome	Effect estimate	95% CI	*p*‐Value	Post hoc power
Graft failure rate	RR = 2.7	0.7‐10.0	0.18	29.1%
Complication rate	RR = 1.5	0.6‐3.7	0.44	11.4%
ACL‐RSI	Cohen's *d* = 0.16	NA	0.27	14.1%
IKDC	Cohen's *d* = 0.21	NA	0.20	21.2%
Tegner	Cohen's *d* = 0.19	NA	0.19	18.5%
SKV	Cohen's *d* = 0.01	NA	0.58	5.0%
Duration of surgery	Cohen's *d* = 0.80	NA	<0.0001	99.2%

*Note*: Relative risks (RR) with 95% CI were calculated for graft failure and complication rates. Continuous outcomes are presented as Cohen's *d* effect sizes. Post hoc statistical power was calculated using two‐sided tests (*α* = 0.05) based on the observed between‐group differences.

Abbreviations: ACL‐RSI, ACL‐Return to Sport after Injury; CI, confidence interval; IKDC, International Knee Documentation Committee; SKV, Subjective Knee Value.

The absolute risk difference for graft failure was 8.2% (13.0% vs 4.8%), corresponding to a relative risk of 2.7 (95% CI: 0.7–10.0) for ACL augmentation compared with ACL reconstruction. Post hoc analysis demonstrated a statistical power of 29.1% for detecting the observed difference (Table 4).

The absolute risk difference for complications was 5.6% (16.7% vs. 11.1%), corresponding to a relative risk of 1.5 (95% CI: 0.6–3.7). Post hoc analysis demonstrated a statistical power of 11.4% for detecting the observed difference.

For ACL‐RSI, IKDC, Tegner and SKV scores, effect sizes were small (Cohen's *d* ranging from 0.01 to 0.21), indicating minimal differences between groups. Corresponding post hoc powers ranged from 5.0% to 21.2%, reflecting limited ability of the study to detect small between‐group differences.

Conversely, the difference in duration of surgery showed a large effect size (Cohen's *d* = 0.80) and a post hoc power of 99.2%, supporting the robustness of this finding.

## DISCUSSION

The results of the current single centre study did not show any statistical difference between ACL augmentation and ACL reconstruction, in terms of subjective clinical scores, graft failure rate, or complication rate, at 3 years post‐surgery. However, tourniquet time was significantly longer in the ACL‐A group compared to the ACL‐R group.

Our two populations were statistically comparable for every preoperative and per operative characteristic. Median age in the ACL‐A and ACL‐R groups was 28 and 24 years, respectively, with no significant difference between groups and was similar to that described in the literature [27, 30]. In particular, age was not specified as an inclusion criterion. There was also no significant difference in median BMI and median tunnel width between the two groups, and the proportion of meniscal lesions was also the same.

The two groups were operated on by the same two senior surgeons using standardised surgical techniques previously described in the literature [4, 6]. Unfortunately, tunnel position could not be collected postoperatively, which is a limitation of our study. However, their intended position is the same in both groups, according to the surgical technique described.

The present study included 60 ACL augmentation procedures, making it one of the largest comparative clinical series evaluating this technique. However, the cohort may still be underpowered to detect significant differences in graft failure rates. Although the augmentation group presented a higher proportion of graft failures compared to standard ACL reconstruction, this difference was not statistically significant. Although no statistically significant difference was observed, the higher proportion of reoperations and graft failures in the augmentation group may still be clinically relevant and should be interpreted cautiously given the limited cohort size. Schneider et al. [26] evaluated ACL augmentation in a cohort of 93 patients and reported good short‐term functional outcomes, with a mean IKDC score of 87 and a low revision rate at 1‐year follow‐up.

The minimum follow‐up in the present study was 3 years, which remains relatively short but exceeds the period during which most graft failures occur after ACL reconstruction [23]. Biological graft remodelling and revascularization are thought to progress substantially during the first two post‐operative years [24], while ligamentization may already be advanced after 1 year regardless of graft type [20]. Despite the theoretical biological advantages of remnant preservation and augmentation, no clinical superiority was observed in the present cohort.

These findings are consistent with the current literature. Wilson et al. [34] reported a graft failure rate of 10.4% and Heusdens et al. [13] reported a rate of 11% at 2‐year follow‐up after ACL repair with internal brace augmentation. Fayard et al. [9] found a 6% failure rate after ACL augmentation using the gracilis tendon combined with an anterolateral procedure. The 13% failure rate observed in our augmentation group therefore remains within the range reported in previous augmentation studies, although higher than the 4.7% observed in our standard ACL reconstruction group. These rates also remain lower than the failure rates historically reported after isolated ACL repair, which may reach 20% [7].

Finally, Cavaignac and al. presented two reviews of the literature [2, 3], showing the same re‐rupture rate for ACL augmentation and reconstruction, at tree years follow up (11.5% and 8.4%, respectively). The collected functional scores were also similar in the two groups (IKDC, KOOS and Tegner scores). This is in accordance with our study, showing no difference in these data.

Functional outcomes observed in the present study were comparable to those reported in the current literature on ACL augmentation procedures. Müller et al. [21] reported a mean ACL‐RSI score of 75 and a mean IKDC score of 85 at 2‐year follow‐up after ACL augmentation. Similarly, Schneider et al. [26] reported a mean IKDC score of 87 at 1 year, while Heusdens et al. [13] and Vermeijden et al. [32] reported mean IKDC scores of 85 and 91, respectively, at 2‐year follow‐up. The functional outcomes observed in our cohort therefore appear consistent with previously published augmentation series.

In contrast, ACL augmentation was associated with a significantly longer tourniquet time compared with standard ACL reconstruction. This may be explained by the additional technical steps required for remnant preservation and tensioning, including careful arthroscopic dissection of the residual ACL fibres before femoral fixation.

The main strength of this study is the similarity between the two groups, the 3 year follow‐up, and the reliability of the surgical procedures. This was a monocentric, comparative study and patients in the two groups were operated on by the same two senior surgeons.

A previous report has suggested that that a lateral extra‐articular procedure with the anterolateral ligament could reduce the failure rate by 54% [12]. In our study, no patient underwent this procedure, and patients with anterolateral ligament reconstruction were excluded in order to decrease the risk of analysis bias. The absence of external procedure may be a limitation to our study. Objective post‐operative laxity evaluation, including pivot‐shift grading and instrumented anterior laxity measurements, was not available in a standardised manner and therefore could not be analysed.

Although no statistically significant differences were observed between groups regarding graft failure and complication rates, and clinical outcomes at 3 years of follow up, post hoc analyses revealed wide confidence intervals and low statistical power for these outcomes. Therefore, the study may have been underpowered to detect potentially clinically meaningful differences, and a type II error cannot be excluded. Consequently, the absence of statistical significance should not be interpreted as evidence of equivalence between ACL augmentation and ACL reconstruction. Larger prospective studies are required to better determine whether the clinical results observed in the ACL‐A group represent true differences or chance findings.

The indication for ACL augmentation was based on intraoperative remnant characteristics according to the principles described by Sherman et al. [27], rather than random allocation. Consequently, patients undergoing augmentation and standard reconstruction did not represent identical injury patterns, and residual selection bias cannot be excluded despite comparable baseline characteristics.

Graft integration and remnant healing were not evaluated using post‐operative MRI. Such imaging evaluation could have provided additional information regarding biological graft maturation and remnant incorporation [31]. The ACL augmentation technique used in this study is different from the ACL repair and ACL remnant conservation technique so it is difficult to compare the results. It has been reported that remnant conservation could improve graft integration, without increasing the rate of cyclops syndrome [15], but this was not associated with the clinical results in our study.

Despite the lack of positive results, ACL augmentation should remain an option for knee surgeons, as either ACL reconstruction or repair [25].

## CONCLUSION

ACL augmentation did not improve post‐operative functional outcomes or decrease graft failure rates at 3‐year follow‐up compared with standard ACL reconstruction, despite a longer tourniquet time and a more technically demanding procedure.

There were higher failure rates and reoperation rates in the ACL augmentation group compared to ACL group; however, this did not reach statistical significance.

## AUTHOR CONTRIBUTIONS

All authors contributed to the study conception and design. Material preparation, data collection and analysis were performed by Corentin Hercé and Nicolas Bougennec. The first draft of the manuscript was written by Corentin Hercé, corrected by Nicolas bouguennec and all authors commented on previous versions of the manuscript. Philippe colombet was one of the main surgeons of our study and the main designer. All authors read and approved the final manuscript.

## FUNDING INFORMATION

The authors have no funding to report.

## CONFLICT OF INTEREST STATEMENT

The authors declare no conflicts of interest.

## ETHICS STATEMENT

Vivalto Santé Institutional Review Board (CERC – IRB). CERC‐VS‐2025‐10‐1.

## Data Availability

Data available on request from the authors.
